# Traumatic False Passage During Nasogastric Tube Insertion and Spontaneous Small Bowel Perforation in a Patient With Vascular Ehlers-Danlos Syndrome: A Case Report

**DOI:** 10.7759/cureus.60063

**Published:** 2024-05-10

**Authors:** Blanche Lee

**Affiliations:** 1 General Surgery, Queen Elizabeth II Jubilee Hospital, Brisbane, AUS

**Keywords:** small bowel resection, connective tissue disorders, nasogastric tube complication, small bowel perforation, vascular ehlers danlos syndrome

## Abstract

Ehlers-Danlos Syndrome (EDS) is a rare connective tissue disorder characterized by mutation in genes that encode or modify collagen. Clinical findings in these patients include skin hyperextensibility, hypermobility of joints, and tissue fragility. Vascular EDS (vEDS) is an autosomal dominant disease typically caused by a mutation in *COL3A1*, which encodes type III collagen. Presenting signs in the majority of vEDS patients include arterial rupture, uterine rupture, and sigmoid colon perforation. In this case report, the author presents an unusual case of spontaneous small bowel perforation and the creation of a traumatic false passage in the parapharyngeal space during a complicated nasogastric tube insertion in a patient with vEDS.

## Introduction

Ehlers-Danlos syndrome (EDS) is a rare hereditary connective tissue disorder that is characterized by tissue fragility, hypermobility of joints, and skin laxity. Historically, the Villefranche Nosology published in 1998 by Beighton et al. classifies EDS into four subtypes [1]. Type IV EDS is also known as the vascular subtype [2]. More recently, Malfait et al. have proposed a revision of the previous EDS classification into 13 subtypes [3]. Mutations in genes that encode for collagen, or collagen-modifying enzymes, have been found in most of these 13 subtypes. This revised classification relies on molecular confirmation of a causative gene and clinical criteria that should raise suspicion for diagnosis. This permits better grouping of subtypes according to causative proteins that function within the same molecular pathway.

This case report will focus on vascular EDS (vEDS). The molecular basis that characterizes vEDS is a defect most often found in the *COL3A1 *gene responsible for the production of the components of type III collagen [3]. Type III collagen is an important structural protein found abundantly in the skin, bowel wall, and blood vessels. vEDS is less commonly caused by a specific amino acid substitution in the *COL1A1* gene that encodes for type I collagen [3]. This substitution also results in vascular fragility that mimics that of typical vEDS caused by the *COL3A1* gene mutation.

The major clinical criteria described by Malfait et al. for the diagnosis of vEDS include arterial or uterine rupture, sigmoid colon perforation in the absence of underlying bowel pathology, a family history of vEDS, or cavernous sinus fistula formation in the absence of trauma [3]. These complications have been found to be the presenting sign in 70% of adults with the *COL3A1* variant [4]. Minor criteria of vEDS include thin, translucent skin, easy bruising, acrogeria, spontaneous pneumothorax, small joint hypermobility, congenital hip dislocation, talipes equinovarus, tendon, and muscle rupture, keratoconus, and a characteristic facial appearance with prominent eyes, thin lips, and a small chin [3].

## Case presentation

A 76-year-old female was brought in by ambulance with significant dyspnoea, nausea, five days of generalized abdominal pain, and three days of obstipation. She had no history of vomiting and had minimal oral intake for three days prior to her presentation. Her medical history was significant for vEDS diagnosed at age 30, jejunal perforation with small bowel resection, duodenal diverticulum, adult-onset small bowel intussusception, volvulus, and perforation requiring surgical repair, diverticulosis, rectal prolapse, procidentia, hysterectomy, cholecystectomy, chronic left hemi-diaphragm elevation, osteoporosis, and a myocardial infarction complicated by a post-infarct ventricular septal defect requiring surgical repair. She was an ex-smoker. Prior to her presentation to the hospital, she was mobilizing independently, lived alone, and was independent with her activities of daily living.

On clinical examination, she looked unwell from the end of the bed. She was afebrile, tachypnoeic, hypotensive, and tachycardic. On auscultation of the lungs there was reduced air entry into the left base. Her abdomen was distended and diffusely tender on palpation with signs of peritonism and involuntary guarding. Blood analysis revealed a leucocytosis of 19x10^9^/L (normal: 4.0-11.0x10^9^/L), an elevated C-reactive protein of 346mg/L (normal: <5mg/L), and an acute kidney injury with a creatinine rise to 95 umol/L (normal: 36-73 umol/L). Her venous blood gas showed the presence of acidaemia with a pH of 7.30 (normal: 7.32-7.43), a pCO_2_ of 65 mmHg (normal: 38-54 mmHg), and a blood lactate level of 2.6 mmol/L (normal: 0.5-2.2mmol/L).

**Figure 1:**
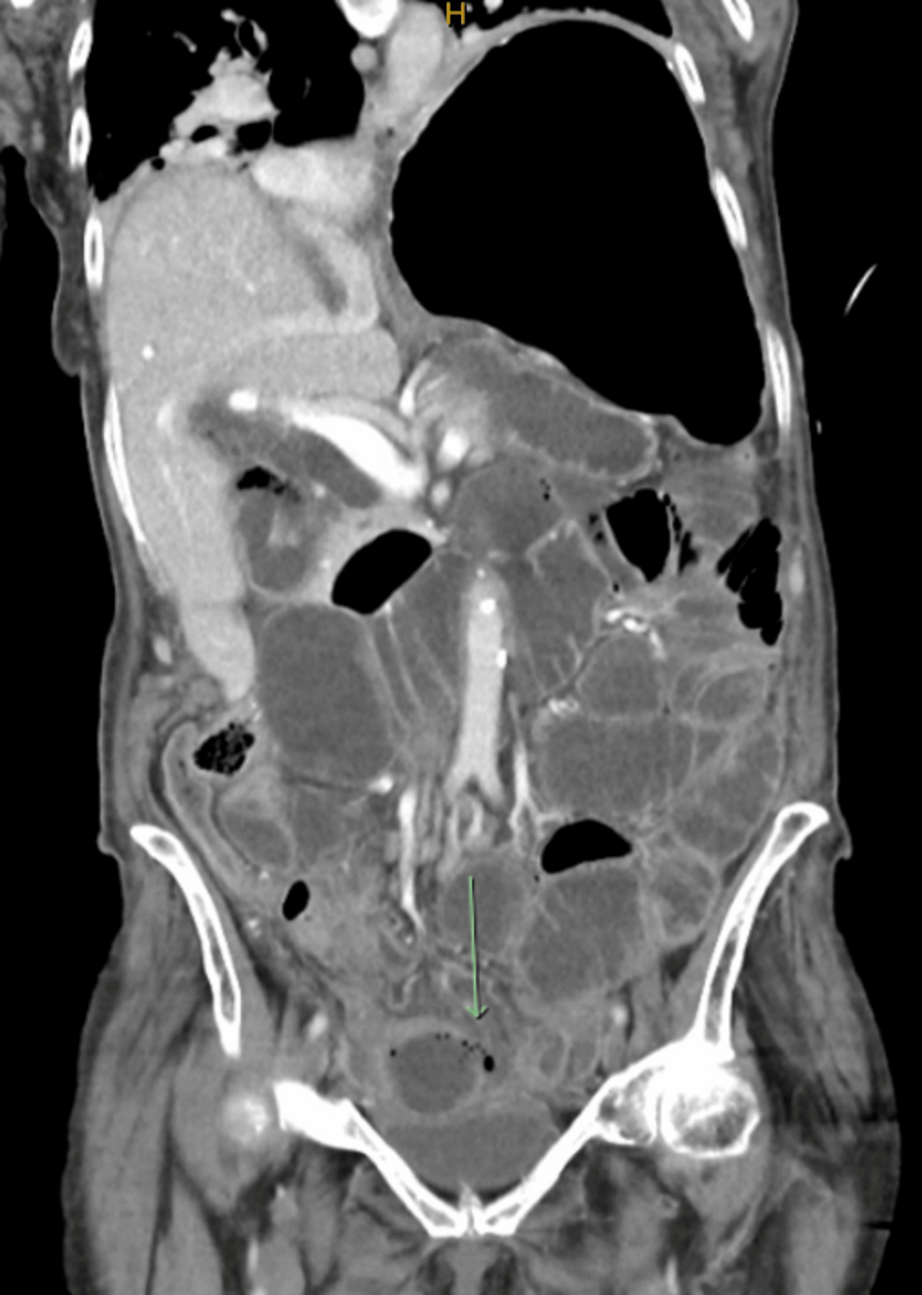
Coronal view of computed tomography (CT) abdomen The image shows dilated small bowel loops with evidence of small bowel perforation (green arrow) and resultant small-volume pneumoperitoneum.

In the emergency department (ED) a chest X-ray (CXR) and computed tomography (CT) scan of her chest and abdomen were performed. The CXR showed compressive atelectasis of the left lung base secondary to severe gastric distention. This was notably worse when compared to her old CXR despite her history of chronic left hemi-diaphragm elevation. The CT scan of the chest was negative for a pulmonary embolism. The abdominal CT scan showed significant dilatation of the small bowel measuring up to 57 mm in diameter. The CT scan was reported as a long segment small bowel obstruction with a mechanical transition point seen in the right pelvic sidewall. There was marked oedema within the distal affected segment of the obstructed small bowel, which was concerning for ischemia. A site of perforation was identified in this distal segment with resultant small volume pneumo-peritoneum and free enteric content within the pelvis (Figure 1).

The patient was resuscitated with intravenous (IV) fluids, commenced on bi-level positive airway pressure for type 2 respiratory failure, given analgesia, commenced on IV antibiotics, and an in-dwelling catheter was inserted for fluid balance monitoring. In the ED there was great difficulty with insertion of a nasogastric tube (NGT) by nursing and medical staff for decompression of the stomach. An NGT was finally inserted on the eighth attempt. The patient was taken to the operating theatre for surgical management with a laparotomy and small bowel resection. Intra-operatively, they found enteric contamination of all four quadrants of the abdomen. The site of perforation was identified in the mid-small bowel on the mesenteric border with pus seen in the adjacent mesentery. There was no clear cause identified for the cause of perforation. There was no adhesiolysis performed. The small bowel was around 1.8 meters in length and was otherwise viable with no evidence of ischemia. There was macroscopic evidence of chronic small bowel changes which was attributed to her history of vEDS. Limited resection of the small bowel was performed with an end-to-end hand sewn double layered anastomosis using a 3-0 polydioxanone suture. The mesenteric defect was closed and a thorough saline wash of the abdomen was performed.

**Figure 2:**
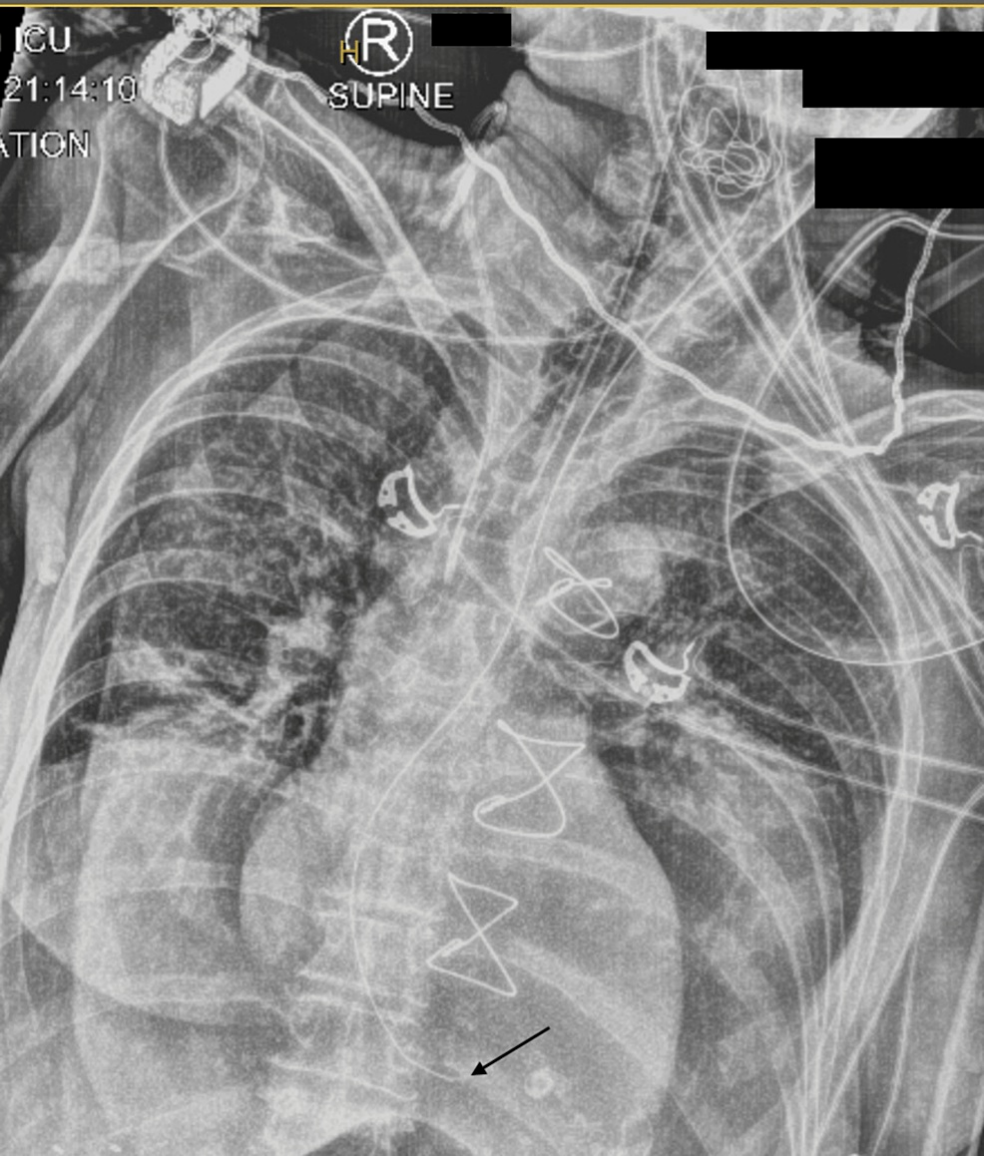
Supine chest X-ray in the intensive care unit The nasogastric tube (NGT) is located at the level of the gastro-oesophageal junction (black arrow). An endotracheal tube, right-sided jugular central venous catheter, electrocardiogram leads, and throat pack are also seen. The patient has sternal wires in situ from prior surgery.

A large hiatal hernia was also noted intra-operatively. It was believed that the NGT placed in ED was not draining properly and was not palpable in the stomach. At the end of the case, a nasogastric tube was re-inserted by the anaesthetic team under direct vision with laryngoscopy and Magill forceps. Unfortunately, an inadvertent laceration to the posterior pharynx was sustained during re-insertion, and gauze packing was placed in the oropharynx to help control bleeding. The patient had a planned admission to the intensive care unit (ICU) post-operatively and remained intubated. Twelve hours after surgery the patient’s abdomen remained distended, she had increasing vasopressor requirements, and staff were unable to aspirate from the NGT. There was concern that the NGT remained improperly sited. A repeat CXR showed the NGT was located at the level of the gastro-oesophageal junction (Figure 2). An attempt was made to advance the NGT further from 45 cm to 65 cm at the nostril. However, this resulted in coiling of the NGT in the oropharynx which was likely caused by the presence of gauze packing. An attempt was made to remove the throat pack, but with attempted manipulation, there was some tugging on the endotracheal tube (ETT). The decision was made to leave the throat pack in place to avoid dislodgement or malpositioning of the ETT.

The following day, an upper endoscopy was performed by a gastroenterologist in the ICU to reposition the NGT. The throat packing was removed, and initially, a paediatric gastroscope was used. This revealed a swollen supraglottic region with patches of hematoma seen around the pharynx. They were unable to intubate the oesophagus due to significant swelling, which resulted in no luminal view. They decided to change to a therapeutic gastroscope that was inserted past the cricopharyngeus muscle under direct vision with a video laryngoscope performed by the intensivist. Despite maximal CO_2_ insufflation, they were unable to visualize the oesophageal lumen. Fresh blood was noted oozing around the nasogastric tube, and the decision was made to abort the procedure. However, the NG tube was able to be advanced slightly further at the nose. A repeat CXR finally showed adequate positioning of the NGT within the large hiatus hernia. Steroids were given for pharyngeal swelling post-procedure. She was successfully extubated the following day. The NGT was left on free drainage with four hourly aspirates, and the patient remained nil by mouth (NBM) with small sips of water for comfort. Upon having small amounts of water orally, she experienced a significant amount of coughing. She was made strictly NBM and then commenced on enteral nasogastric feeds as the NGT was now well located. A speech pathologist was consulted for a swallow assessment. The assessment confirmed that the patient should remain nil by mouth (NBM) due to a high risk of aspiration secondary to severe oropharyngeal dysphagia. A video fluoroscopic swallowing study (VFSS), also known as a modified barium swallow, was performed.

**Figure 3:**
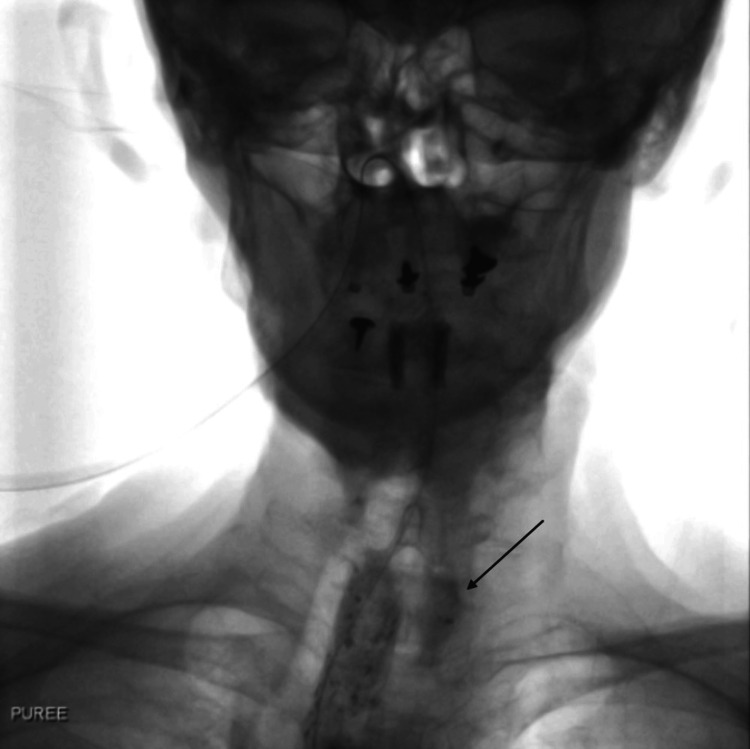
Videofluoroscopic swallowing study image The image shows the filling of a left parapharyngeal pouch (black arrow).

The VFSS revealed contrast filling of a left parapharyngeal pouch, which likely represented a traumatic false passage (Figure 3). It also showed evidence of aspiration when the patient swallowed thin fluids. The differential diagnosis was a pre-existing pharyngeal pouch also known as a Zenker’s diverticulum. This seemed less likely as the patient had no symptoms of dysphagia, regurgitation, or history of aspiration prior to admission.

**Figure 4:**
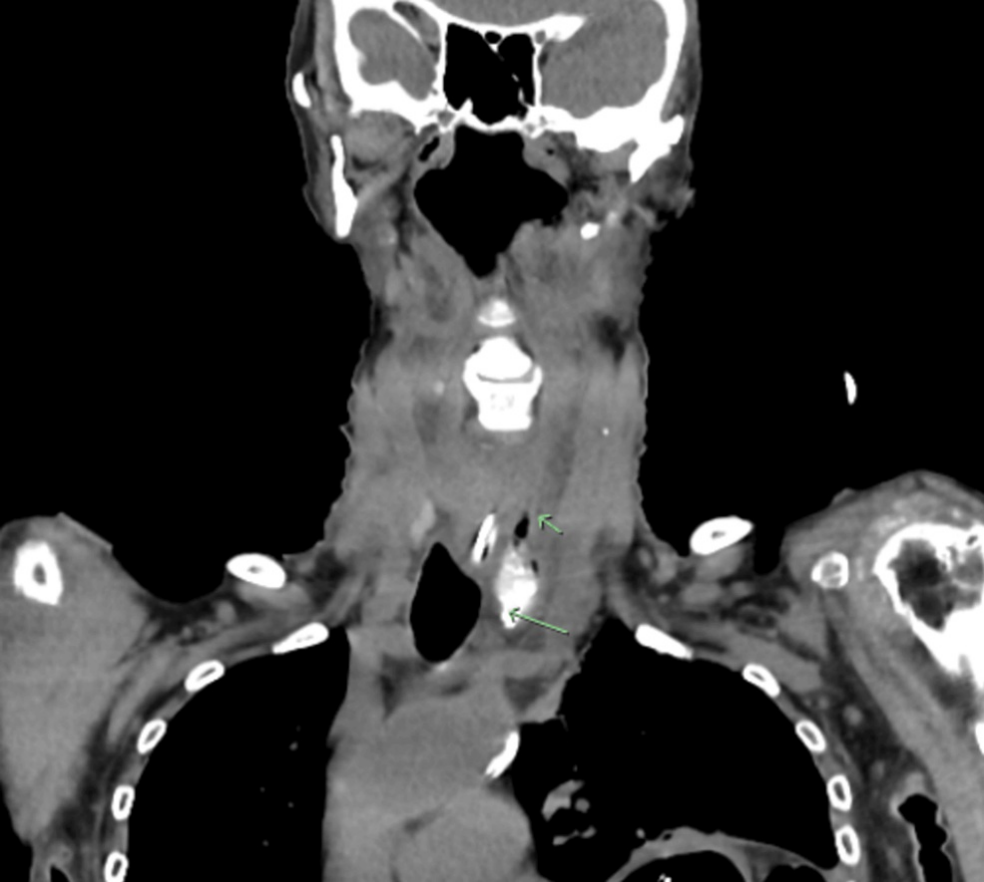
Coronal view of a non-contrast computed tomography of the neck which confirms a left parapharyngeal collection with free gas (green arrows) and pooling of the previously swallowed modified barium contrast material.

A subsequent non-contrast CT was performed of the neck which showed evidence of free gas and pooling of the modified barium contrast material at the base of the neck (Figure 4). The internal opening of the false passage appeared to be located at the level of the cricoid cartilage and extended down to approximately C7. 

The ear, nose, and throat (ENT) team was consulted for this pharyngeal pouch. They advised the patient to remain NBM for a further week and then to perform a repeat VFSS. The repeat VFSS showed no contrast in the left parapharyngeal space where the false passage was noted previously. The patient made good progress post-operatively. As she regained bowel function, her diet was gradually upgraded with acknowledgment of the risk of oral feeding. She was subsequently discharged 13 days after her surgery. She remained well with no complications at two months when she was seen in the surgical outpatient department. An interval CT scan of the neck at two months with the ENT team also showed no evidence of a left parapharyngeal pouch, and the patient had no further issues with swallowing. Subsequent histopathology of the small bowel showed a site of perforation with associated necrosis, and the presence of serositis. The mucosa was unremarkable. The margins did not appear significantly devitalized and there was no sign of malignancy.

## Discussion

The aetiology of small bowel perforation includes trauma, bowel ischemia, bowel obstruction, and neoplasms. Less common aetiologies of small bowel perforation include connective tissue disorders, inflammatory bowel conditions, or infections that weaken tissue. Duodenal perforations can be associated with peptic ulcer disease, vasculitis, and non-steroidal medication. Perforation of the gastrointestinal tract occurs in roughly 15% of vEDS patients identified with the *COL3A1* variant [4]. The most common gastrointestinal manifestation of vEDS is spontaneous sigmoid colon perforation in the absence of any underlying bowel pathology, such as diverticulosis [3]. Cases of spontaneous perforation of the small bowel and stomach have also been reported, although much less frequently [2]. However, gastrointestinal rupture was rarely found to lead to death [2]. The majority of deaths in this patient population resulted from vascular complications such as arterial rupture in the thorax and abdomen [3]. The median survival in studied vEDS patients was 50 years [4].

Surgical intervention for spontaneous gastrointestinal perforation is often required as a life-saving measure. Colonic perforation was most often managed with a partial colectomy, formation of an end colostomy, and then interval reversal of the colostomy with re-anastomosis of the bowel was performed [2,4]. Patients were less commonly treated with a partial colectomy and immediate end-to-end re-anastomosis [4]. In rare cases, patients underwent total colectomy for recurrent perforations [2]. There is sparse literature on the best approach for the management of spontaneous small bowel perforations in vEDS patients. One case report also successfully managed a small bowel perforation with resection and immediate entero-enteric anastomosis in a patient with vEDS [5]. Another case report performed a jejunal resection with a functional end-to-end anastomosis, which was complicated by an anastomotic leak found at the entry hole of the linear stapler used [6]. A further jejunal resection was performed of the affected segment, and a single-hole jejunostomy was performed, and this was subsequently reversed two months later [6].

The increased risk of complications from surgery in this patient population is related to general tissue and vessel fragility. These complications include poor wound healing, dehiscence of the wound, evisceration, fistula formulation, or recurrent bowel or arterial injury [4]. Those who survive surgery may have recurrent bowel perforations with an unpredictable time course. It is unclear whether vEDS increases the risk of iatrogenic injury with invasive procedures such as nasogastric tube insertion or colonoscopy, but this would appear plausible. Iatrogenic perforation in this patient cohort during colonoscopy has also been reported in the literature [7].

## Conclusions

Spontaneous small bowel perforation is an uncommon presentation in patients with vEDS. Surgical intervention is often a necessary and life-saving measure for those who present with spontaneous small bowel perforation. This is the first report of a traumatic false passage secondary to NGT insertion in a vEDS patient. Awareness and understanding of the underlying pathophysiology of vEDS should prompt extra care when clinicians are performing invasive procedures such as nasogastric tube insertion or colonoscopy to avoid iatrogenic complications. There should be early consideration of inserting an NGT under vision with video laryngoscopy in this patient cohort should initial attempts prove challenging.
